# Targeting MYC and BCL2 by a natural compound for “double-hit” lymphoma

**DOI:** 10.1002/hon.3010

**Published:** 2022-05-05

**Authors:** Xiaoqian Liu, Senlin Xu, Jiawei Zhang, Mingjie Fan, Jun Xie, Bingfeng Zhang, Hongzhi Li, Guohua Yu, Yinghui Liu, Yuanfeng Zhang, Joo Song, David Horne, Wing C. Chan, Xiaoxia Chu, Wendong Huang

**Affiliations:** 1 Department of Hematology, The Affiliated Yantai Yuhuangding Hospital of Qingdao University, Yantai, Shandong, China; 2 Molecular and Cellular Biology of Cancer Program & Department of Diabetes Complications and Metabolism, Beckman Research Institute, City of Hope, Duarte, California, USA; 3 Department of Molecular Medicine, City of Hope National Medical Center, Duarte, California, USA; 4 Department of Pathology, The Affiliated Yantai Yuhuangding Hospital of Qingdao University, Yantai, Shandong, China; 5 Department of Pathology, City of Hope National Medical Center, Duarte, California, USA

**Keywords:** Bcl2, berbamine, c-Myc, cancer treatment, double-hit lymphoma

## Abstract

Concurrent translocations of *MYC* and *BCL2* lead to abnormal expression of both oncoproteins, which contribute to the aggressive clinical characteristics of double-hit lymphoma (DHL). An effective therapy for DHL remains an unmet clinical need. In this study, we showed that both Ca^2+^/calmodulin-dependent protein kinase II δ (CAMKIIδ) and *γ* (CAMKIIγ) were highly expressed in DHL. Both isoforms of CAMKII stabilize c-Myc protein by phosphorylating it at Ser62, increase BCL2 expression, and promote DHL tumor growth. Inhibition of CAMKIIδ and CAMKIIγ by either berbamine (BBM) or one of its derivatives (PA4) led to the down regulation of c-Myc and BCL2 proteins. BBM/PA4 also exhibited anti-tumor efficacy in DHL cell lines and NSG xenograft models. Altogether, CAMKIIδ and CAMKIIγ appear to be critical for DHL tumor development and are promising therapeutic targets for DHL.

## 1 | INTRODUCTION

In the most recent WHO classification of lymphoid tumors,^1^ a unique category was established for both “double-hit” lymphoma (DHL) and “triple-hit” lymphoma (THL). These two B-cell lymphomas were separated from diffuse large B cell lymphoma (DLBCL) and designated in a new category called high-grade B-cell lymphoma (HGBL) with translocations of *MYC, BCL2,* and/or *BCL6*. DHL shows highly aggressive clinical features in advanced stages, rapid progression, and a propensity to involve the central nervous system. Patients with this subtype of lymphoma have poor outcomes when treated with the conventional immune-chemotherapy Rituximab with cyclophosphamide, doxorubicin hydrochloride, vincristine, and prednisolone (RCHOP).^2^ Therefore, to better understand the molecular pathogenesis of DHL and develop new therapeutic approaches are urgent.

Concurrent translocations of *MYC* and *BCL2* lead to abnormal expression of both oncoproteins. Effective strategies to treat DHL may involve targeting MYC and/or BCL2.^3–5^ Previously, we have been developing approaches to target MYC indirectly.^6,7^ In our studies, CAMKIIγ stabilizes c-Myc by directly phosphorylating Serine 62 (S62). When CAMKIIγ is inhibited with a natural compound called berbamine (BBM), this process can be suppressed, leading to c-Myc protein degradation. Accordingly, BBM suppressed the growth of T cell lymphoma both in vitro and in vivo.^6^ Based on the BBM chemical structure, we have developed several new derivatives. One derivative, termed PA4, inhibited both CAMKIIγ and CAMKIIδ, and showed more potent activity than BBM in killing lymphoma cells.

ABT199 (Venetoclax) is a highly selective inhibitor of BCL2.^8^ It has been approval by the FDA for treating either chronic lymphocytic leukemia or small lymphocytic lymphoma. Moreover, several studies suggest that ABT199 is a promising drug for treating DHL.^4,9^ Low concentrations of ABT199 effectively induced cell apoptosis in “double-protein expression” lymphoma with *MYC* and *BCL2* rearrangements.

Given this landscape, we hypothesize that a combination of BBM/PA4 with ABT199 will exhibit a synergistic effect in DHL. We also demonstrated PA4 as a more potent inhibitor of cancer cell growth than BBM. According to our results, treatment with the PA4 effectively inhibited both c-Myc and BCL2, which impaired both STAT3 and NFAT signaling pathways. We contextualize these results by discussing the relationship between CAMKII and downstream targets in DHL.

## 2 | METHODS

### 2.1 | Patient specimens

Paraffin sections of DLBCL patients (*n* = 28) and DHL patients (*n* = 10) were obtained from the Department of Pathology Qingdao University Medical College Affiliated Yantai Yuhuangding Hospital. All the collections of samples were carried out with informed consent according to the Declaration of Helsinki. Ethical approval was provided by the Ethics Committee of Qingdao University Medical College Affiliated Yantai Yuhuangding Hospital.

### 2.2 | Cell lines and reagents

Four DHL cell lines (NU-DHL-1, SU-DHL-6, OCI-LY-19 and DOGKIT) and a Burkitt cell lymphoma cell line (Jiyoye) were employed. NUDHL-1, SU-DHL-6 and OCI-LY-19 were maintained in IMDM (Corning) with 10% FBS (Omega Scientific). DOGKIT and Jiyoye were maintained in RPMI 1640 (Corning) with 10%FBS. All the cells were cultured at 37°C in a 95% air, 5% CO2 humidified incubator.

BBM, PA4, and ABT199 (Millipore Sigma) were dissolved in stock solutions with dimethyl sulfoxide (DMSO) for treatment at IC50 concentrations if not otherwise indicated. All antibodies and other reagents used are listed in Table S1.

### 2.3 | IHC analysis

Immunohistochemical (IHC) staining for patients’ samples was described in supplementary information. Tumors harvested from mice were fixed in 4% PBS-buffered formaldehyde solution, dehydrated, and embedded in paraffin. Paraffin-embedded samples were sectioned and processed for hematoxylin and eosin (H&E) staining and IHC staining. The staining was performed by the Pathology Core of City of Hope Medical Center.

### 2.4 | MTS ASSAYS

Cells cultured in 96-well plates and exposed to different concentrations of BBM/PA4/ABT199 for 24 h. Cell viability was determined by CellTiter 96 Aqueous Cell Proliferation Kit (Promega). IC_50_ was defined as the drug concentration that induced a 50% viability decrease.

### 2.5 | Gene knockout using the CRISPR/Cas9 system

Single guide RNA (sgRNA) sequences targeting exon seven of human CAMK2D and exon one of CAMK2G were designed using the CRISPR on-line design tool (www.genome-engineering.org/crispr; Table S2). The designed sequences were cloned into pSpCas9(BB)-2A-GFP (PX458, Addgene) and validated by Sanger sequencing (Figure S3A). The sgRNA-containing pX458 was electroporated into SU-DHL-6 cells with the Cell Line Nucleofector Kit V (Lonza) following manufacturer instructions. Forty-8 hours later, single green fluorescent protein-positive (GFP+) cells were sorted by FACS into 96-well plates and expanded to individual clones. Single clones were screened by qPCR with two pairs of primers (Table S2) covering the cutting site of CAMK2D and CAMK2G. Compared with wide type (WT), knockout clones showed a different melting temperature were selected (Figure S3B). Western Blot analysis was performed to confirm knockout.

### 2.6 | Xenograft model

Murine experiments were in accordance with a protocol approved by the Animal Care and Use Committee in City of Hope (Duarte, CA). 3 × 10^6^ SU-DHL-6 cells suspended in RPMI 1640 were injected subcutaneously in the right flank of female NSG mice. After the xenografted tumors reached ~30 mm^3^, mice were randomized to 2 groups1: PBS control, *n* = 8^2^; treated with 5 mg/kg PA4, *n* = 10. Mice were treated daily via oral gavage for 12 days. Tumors and body weight were measured three times a week with Vernier calipers, and the tumor volume was calculated as length × width^2^/2. Mice were euthanized, and tumor tissues were collected after the 12th day of treatment.

### 2.7 | Statistical analysis

Difference between two groups was calculated by Mann-Whitney *U* test or Studenťs *t*-test (two-sided). For intercomparison of more than 2 groups, a one-way ANOVA followed by a post-hoc test was applied. To assess the association between c-Myc and CAMKIIγ, Pearson correlation analysis was used. *p* values < 0.05 were considered statistically significant. In the figures, changes are noted using *p < 0.05 and **p < 0.01. Statistical analysis was conducted using SPSS or GraphPad Prism.

## 3 | RESULTS

### 3.1 | CAMKIIδ, CAMKIIγ, p-c-Myc (S62), c-Myc and BCL2 expressions were frequently observed in DLBCL and DHL patients

**FIGURE 1:**
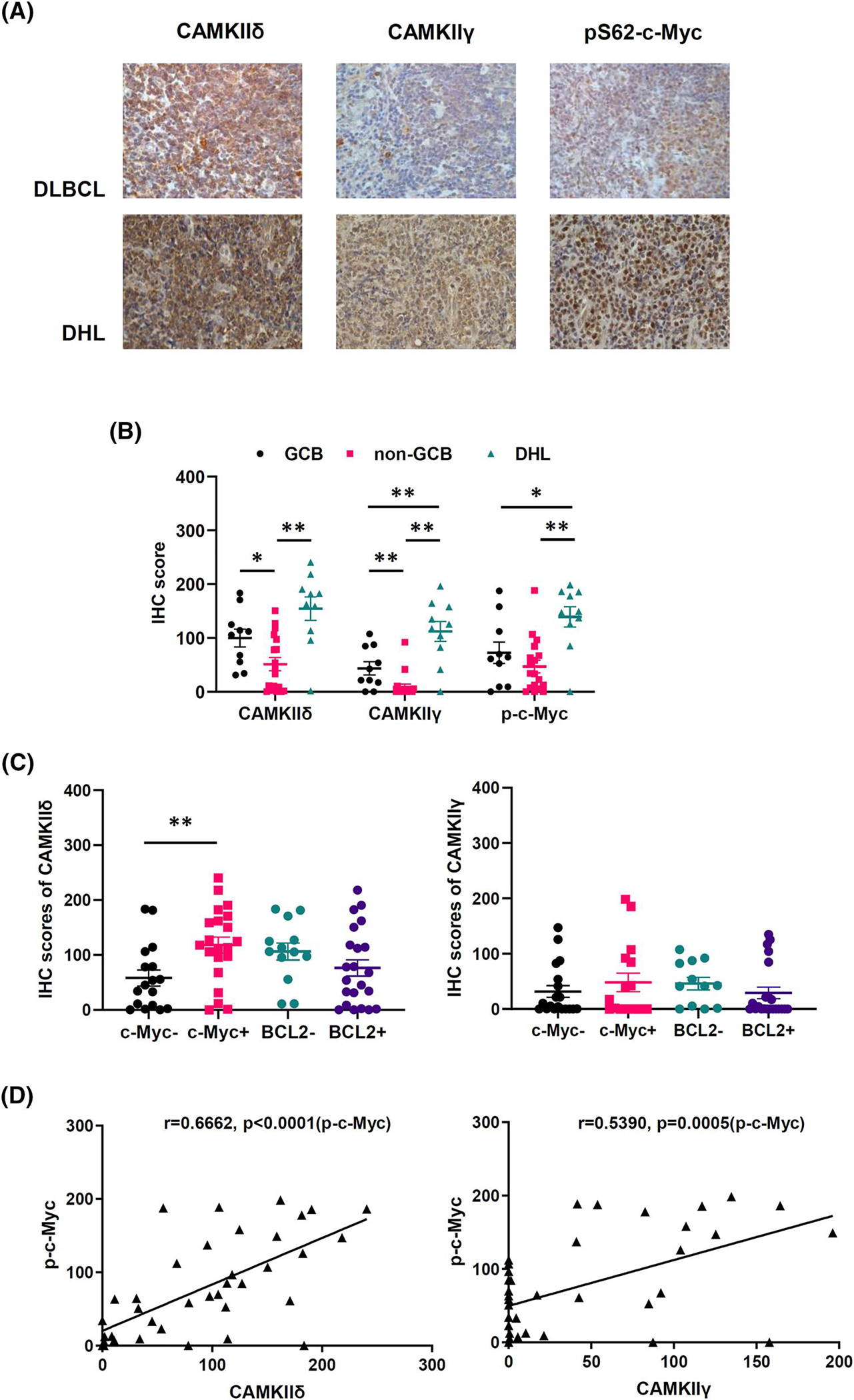
Positive correlations of expressions detected by Immunohistochemical (IHC) staining among Ca^2+^/calmodulin-dependent protein kinase II δ (CAMKIIδ), Ca2+/calmodulin-dependent protein kinase II γ(CAMKIIγ), c-Myc and pS62-c-Myc in diffuse large B cell lymphoma (DLBCL) and double-hit lymphoma (DHL) patients. (A), Representative images of IHC staining of CAMKIIδ, CAMKIIγ, and pS62-c-Myc in DLBCL and DHL tissues. (B), IHC scores in subgroups of clinical cases subgrouped by the cell-of-origin (COO). (C), IHC scores of CAMKIIδ and CAMKIIγ sybgrouped by c-Myc or BCL2 expression. (D), Correlations between CAMKIIδ (left) or CAMKIIγ (right) and pS62-c-Myc expression in human DLBCL and DHL samples. Data are shown as mean ± SEM and compared by unpaired Studenťs t-test. Correlations are shown using Pearson’s r and significance determined using a Spearman correlation. *p < 0.05; **p < 0.01

First the expression levels of five proteins–CAMKIIδ, CAMKIIγ, p-c-Myc (S62), c-Myc and BCL2 were assessed across clinical samples of DLBCL and DHL by immunohistochemistry (IHC). Among the 38 cases, IHC clearly detected expression of all proteins with variable frequency and intensity (Table 1). CAMKIIδ was frequently and highly expressed (IHC score≥100) in both DLBCL cases (12/28, 43%) and DHL cases (8/10, 80%; Figure 1A). All CAMKIIδ positive cases exhibited a cytoplasmic expression pattern. The expression pattern of CAMKIIγ was similar to CAMKIIδ with a weaker staining intensity and lower positive cell rate (Figure 1A,B). In different subgroups of DLBCL, both CAMKIIδ and CAMKIIγ were significantly higher in the germinal center B-cell (GCB) and the DHL subgroups compared to the non-GCB group. To investigate the association between CAMKII and c-Myc, patients were separated into Myc-positive (Myc+) and Myc-negative (Myc−) groups (cutoff 40%). Myc+group exhibited a higher CAMKIIδ staining score than Myc-group (Figure 1C left). Pearson correlation analysis showed a positive correlation between expression of both CAMKIIδ/γ and p-c-Myc (S62), c-Myc (Figure 1D). We also compared CAMKIIδ/γ in BCL2-positive (BCL2+) and BCL2-negative (BCL2−) groups (cutoff 50%), but no significant difference was found (Figure 1C right).

### 3.2 | BBM or PA4 effectively inhibited DHL cell growth and induced cell apoptosis

**FIGURE 2:**
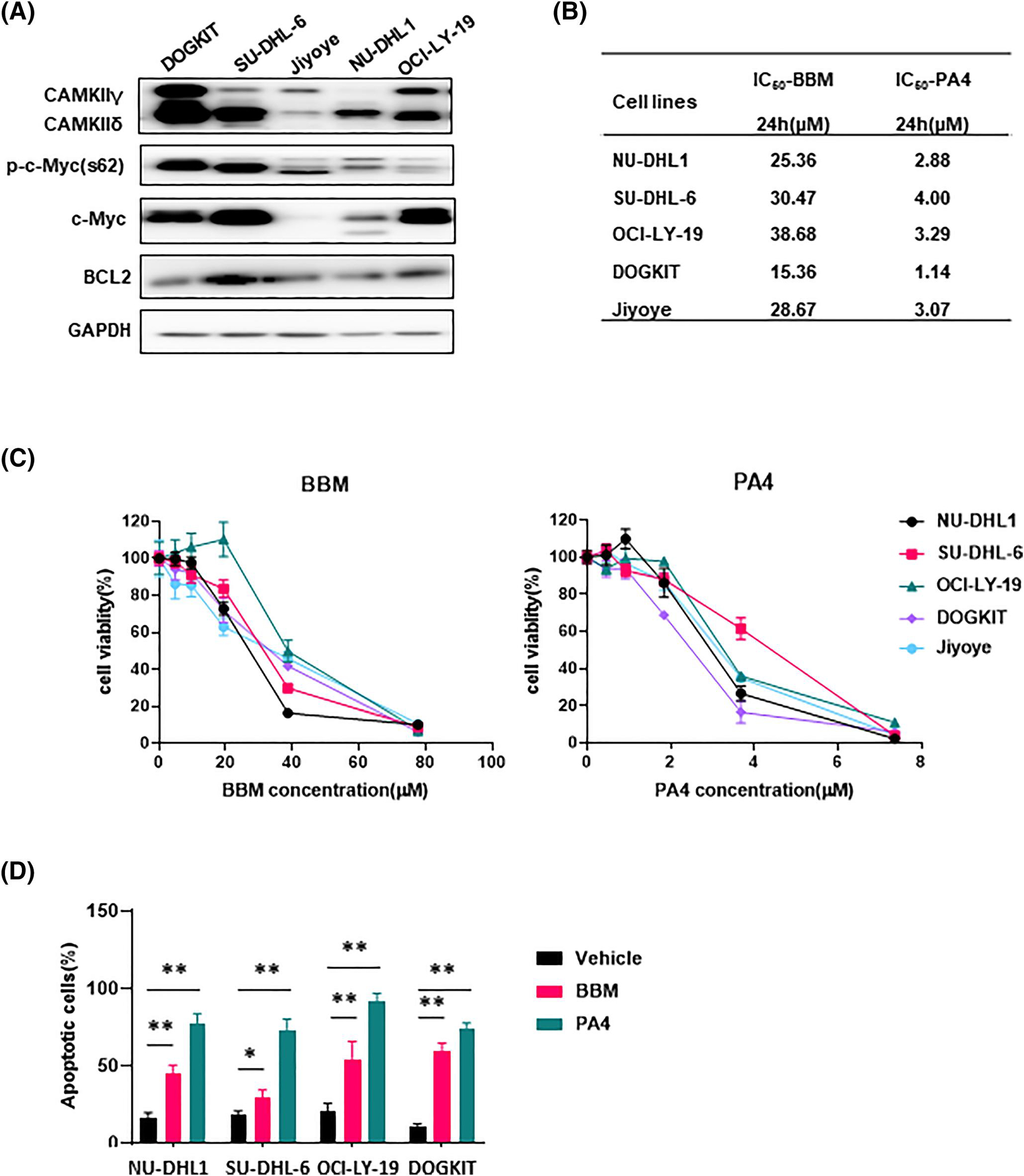
Characteristics and inhibitory effect of BBM, PA4 and ABT199 on four double-hit lymphoma (DHL) cell lines. (A), Western blot analysis of Ca2+/calmodulin-dependent protein kinase II δ (CAMKIIδ), Ca2+/calmodulin-dependent protein kinase II γ (CAMKIIγ), phosphorylated c-Myc (ser62), c-Myc, and BCL2 in four DHL cell lines (DOGKIT, SU-DHL-6, NU-DHL1, and OCI-LY-19). Jiyoye is a Burkitt lymphoma cell line used here as a comparison. (B), IC_50_ concentrations at 24 h of BBM and PA4 for four DHL cell lines and Jiyoye determined by MTS assay. (C), Cell viability determined by MTS assay in DHL cell lines. DHL cell lines were treated with either BBM or PA4 at increasing concentrations. Cell viability was determined by MTS assay at 24 h and normalized to vehicle control. (D), Apoptosis induced by either BBM (20 μM) or PA4 (2 μM) when the treatments were applied to cell lines for 24 h. Cells were stained with Annexin V and DAPI and then evaluated on BD Fortessa cytometer. The collected data were analyzed by FlowJo software. Data are shown as mean ± SEM and significance are determined by unpaired Studenťs *t*-test. *p < 0.05 compared to vehicle; **p < 0.01 compared to vehicle

The expression of c-Myc and CAMKIIδ/γ in four DHL cell lines was examined compared to Burritťs lymphoma cell line Jiyoye (Figure 2A). All four DHL cell lines expressed higher levels of CAMKIIδ/γ, c-Myc, and BCL2. We first determined the effects of BBM and PA4 on cell growth and apoptosis. Both BBM and PA4 inhibited cell growth in a dose-dependent manner (Figure 2B,C), while PA4 possessed a much lower IC_50_ concentration (Figure 2B), suggesting a more potent inhibitor of proliferation.

Cell apoptosis was measured in DHL cell lines after 24-h treatment. Approximately 72.67%–91.57% of cells treated with PA4 were positive for Annexin V; whereas the positive rates were limited to 30%–50% treated with BBM (Figure 2D). These results suggest that PA4 is more effective than BBM at inducing apoptosis.

**FIGURE 3:**
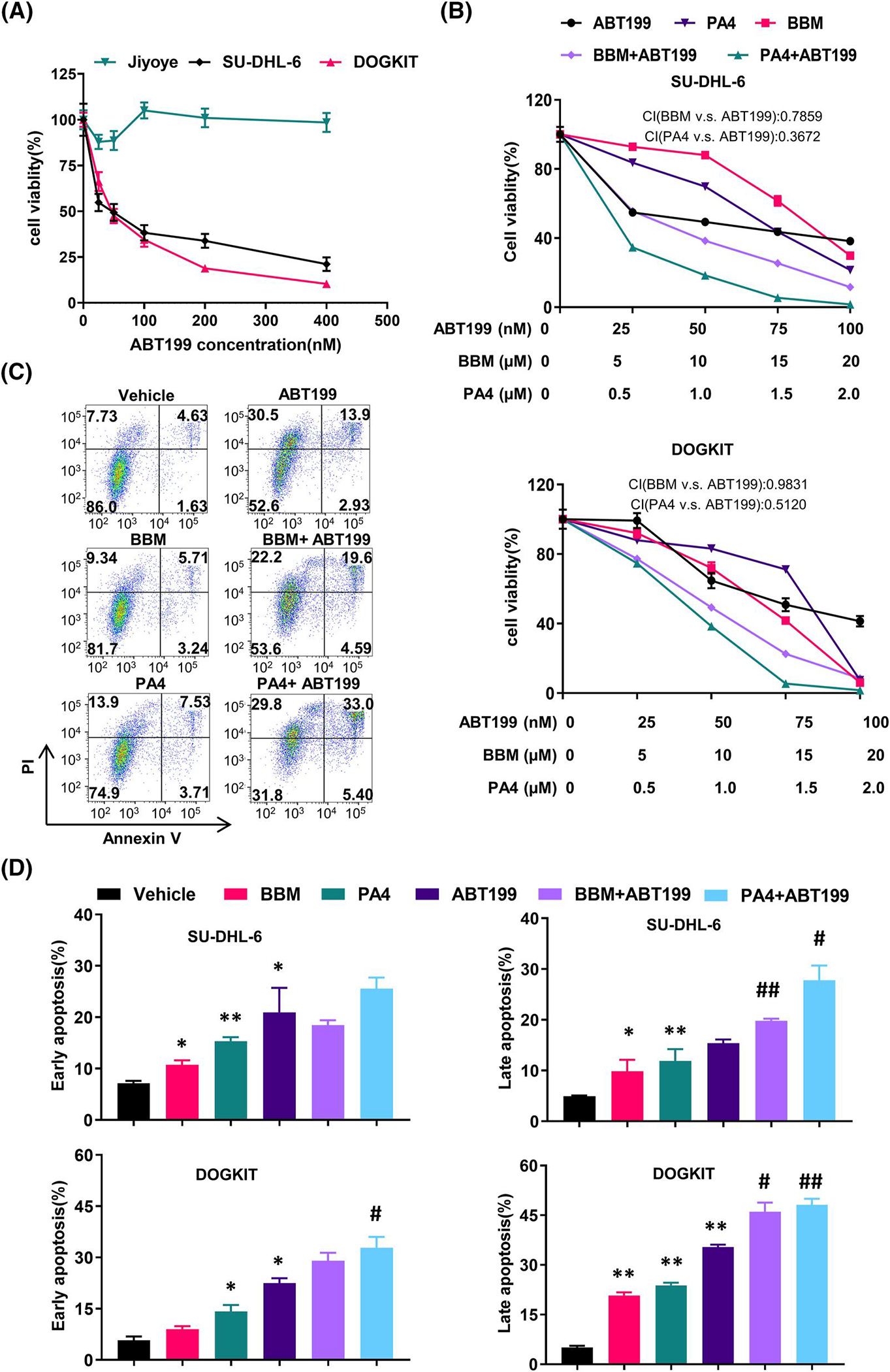
Synergistic effect of BBM/PA4 with ABT199 on DOGKIT and SU-DHL-6. (A), IC_50_ concentrations of ABT199 for double-hit lymphoma (DHL) cell lines determined by MTS assay. (B), Synergistic effects of treatments in DOGKIT and SU-DHL-6. DOGKIT and SU-DHL-6 were either treated with an individual agent (BBM, PA4, or ABT199) or with two of these agents (ABT199 + X) at increasing concentrations. Cell viability was determined by MTS assay at 24 h and normalized to vehicle control. (C), Apoptotic cells 8 h after combined treatment analyzed by Flowjo. D, Early and late apoptosis induced by ABT199 (50 nM), BBM (10 μM), PA4 (1 μM), BBM (10 μM) + ABT199 (50 nM), or PA4 (1 μM) + ABT199 (50 nM). Data are shown as mean ± SEM and significance are determined by unpaired Studenťs *t*-test. *p < 0.05 compared to vehicle; **p < 0.01 compared to vehicle; #*p* < 0.05 compared to ABT199; ##*p* < 0.01 compared to ABT199

As a selective inhibitor of BCL2, ABT199 is a promising drug for DHL. ABT199 inhibited cell growth of DOGKIT and SU-DHL-6 (Figure 3A). Synergistic effects of two different combinations: (1) BBM + ABT199 and (2) PA4 + ABT199 were explored. CI values were calculated to determine the synergistic effects based on previous literature.^10^ We observed a low synergistic effect for BBM + ABT199 (CI_50_ = 0.7978 in SU-DHL-6). In contrast, a high synergistic effect was observed for PA4 + ABT199 (CI_50_ = 0.3672 in SU-DHL-6; CI_50_ = 0.5120 in DOGKIT; Figure 3B). Neither of the combinations displayed a clear synergistic effect on cell apoptosis (Figures 3C,D). Compared to ABT199 treatment alone, a slightly higher number of early apoptotic cells in DOGKIT BBM + ABT199 combination and higher percentage of apoptotic cells in SU-DHL-6 BBM + ABT199 and PA4 + ABT199 combinations were observed.

### 3.3 | PA4 bound to both CAMKIIγ and CAMKIIδ and displayed higher affinity compared to BBM

**FIGURE 4:**
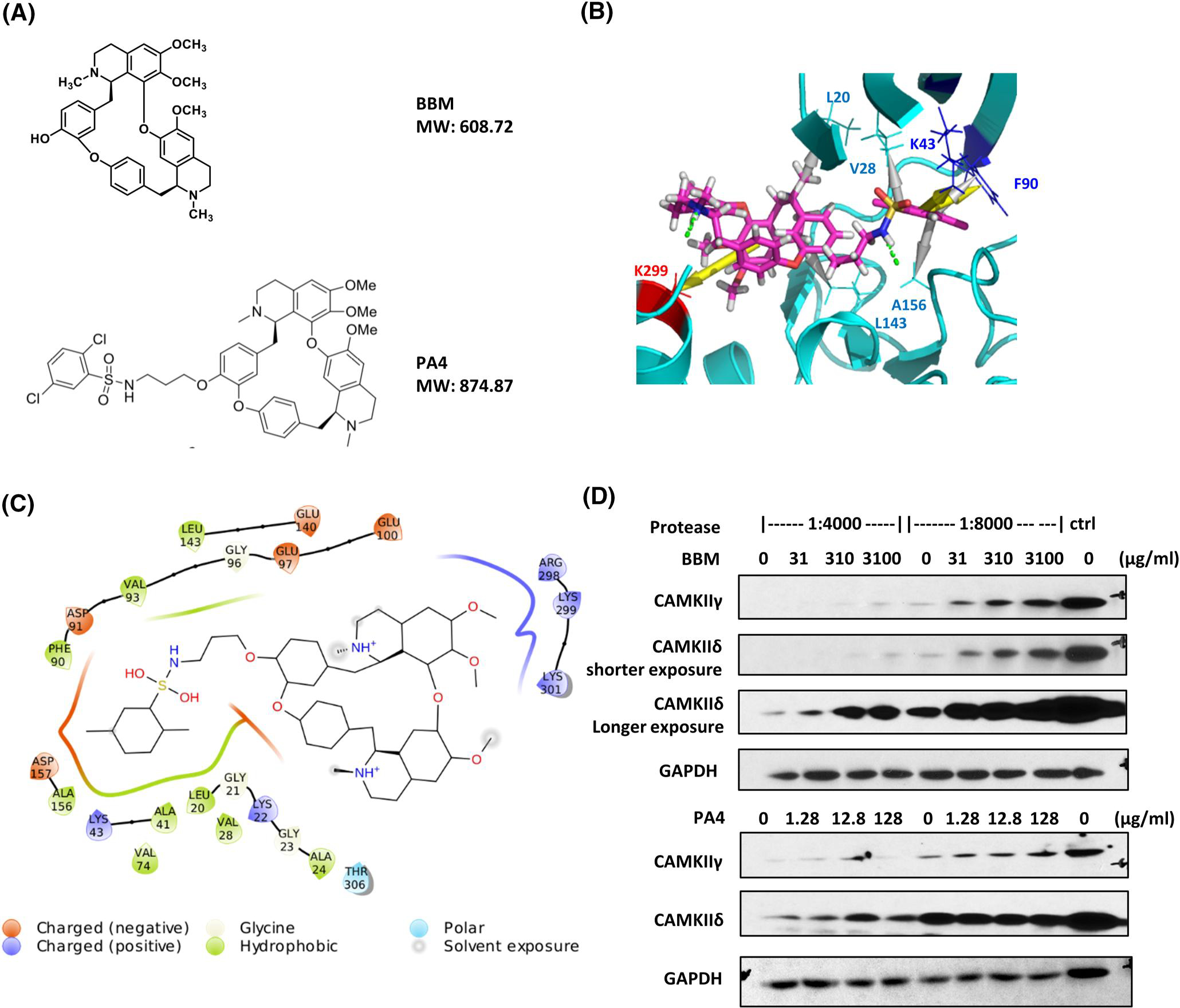
PA4 binds to Ca^2+^/calmodulin-dependent protein kinase II γ (CAMKIIγ) and Ca^2+^/calmodulin-dependent protein kinase II *δ* (CAMKIIδ) and shows higher affinity compared to BBM. (A), Molecular weights and constructs of BBM and PA4. (B), Interactions of PA4 at the ATP-binding pocket of CAMKIIγ. The hydrogen bonds are displayed as green dots, while the hydrophobic interaction pairs are shown as gray arrows. Π-Interaction pairs are depicted as yellow arrows. (C), Two-dimensional interaction diagram of PA4 bound to the ATP-binding pocket of CAMKIIδ. (D), DARTS analysis was performed to identify potential protein targets for BBM and PA4. The cell lysate of SU-DHL-6 was incubated with increasing concentrations of either BBM or PA4 at room temperature for 30 min. Then a protease solution was added into the mixtures to digest the protein. Proteins in the digested products were detected with western blot analysis

The structures of BBM and PA4 are shown in Figure 4A. We previously identified that BBM can bind and inhibit CAMKIIγ by targeting its ATP binding pocket.^7^ Here, docking between PA4 and CAMKII was performed with the Glide docking method. PA4 also displayed the capacity to target the ATP binding pocket of both CAMKIIγ and CAMKIIδ (Figures 4B,C).

To probe binding of the compounds to CAMKIIδ/γ via in vitro experiments, DARTS analysis was performed (Figure S2). Cell lysate was incubated with increasing concentrations of either BBM or PA4; then protease solution was added to digest the protein. The physical binding of the compounds to both CAMKIIγ and CAMKIIδ can protect the proteins from proteolysis in the assay, resulting in higher amounts of undigested proteins detected by western blot (Figure 4D). PA4 exhibited a much lower effective concentration in protecting CAMKIIδ/γ from proteolysis.

### 3.4 | BBM or PA4 inhibits phosphorylation of CAMKIIγ/δ, leading to the instability of c-Myc protein and downregulation of other oncogenic pathways

**FIGURE 5:**
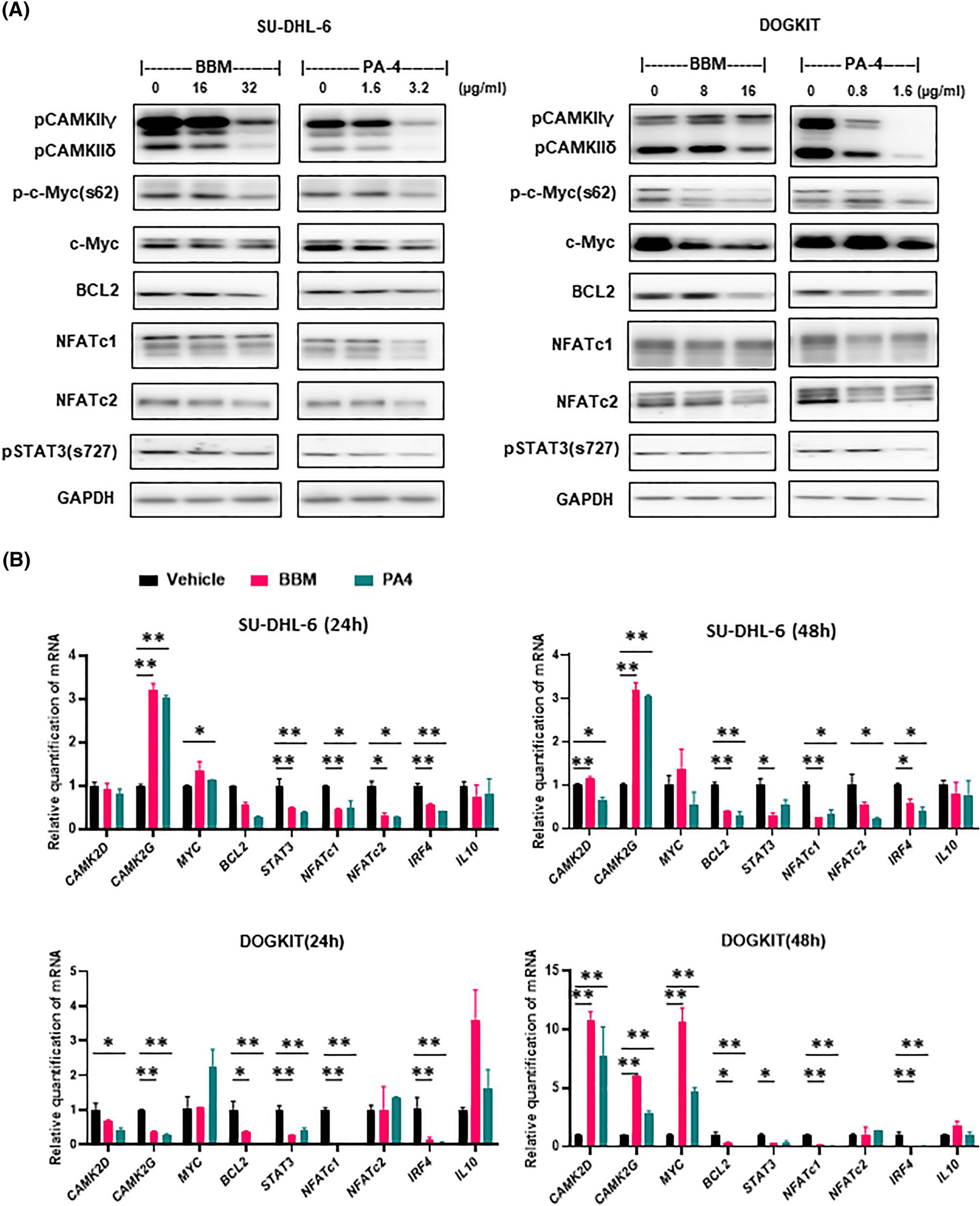
SU-DHL-6 and DOGKIT treated with BBM and PA4. (A), Western blot analysis of CAMKII and potential targets in SU-DHL-6 and DOGKIT treated by BBM or PA4 for 24 h at increasing concentrations. (B), mRNA levels of SU-DHL-6 and DOGKIT treated by BBM (30 μM for SU-DHL-6 and 15 μM for DOGKIT) or PA4 (3 μM for SU-DHL-6 and 1.5 μM for DOGKIT). Data are shown as mean ± SEM and significance are determined by unpaired Studenťs *t*-test. *p < 0.05; **p < 0.01

Furtherly, downstream molecular pathway alterations after BBM or PA4 treatment were assessed. Western blot showed CAMKIIγ and CAMKIIδ as well as total c-Myc and p-c-Myc (S62) were significantly downregulated by either BBM or PA4 in a dose dependent manner (Figure 5A). Our previous studies demonstrated that CAMKIIγ stabilized c-Myc by directly phosphorylating it at S62.^6,11^ The data from DHL cells suggests that the c-Myc is affected by the status of both CAMKIIγ and CAMKIIδ similarly.

Compensatory upregulation of mRNA was observed in *CAMK2G* and *CAMK2D* of DOGKIT as well as *CAMK2G* of SU-DHL-6. Similarly, *MYC* transcription was compensatory increased after long-term exposure to PA4 in DOGKIT (Figure 5B).

Other important genes related to DLBCL growth, such as STAT3 and NFAT pathway^12,13^ are also determined. Here, treatment of DHL cell lines with either BBM or PA4 decreased STAT3 activation and reduced both NFATc1 and NFATc2 levels (Figure 5A). Both *STAT3* and *NFAT* mRNA significantly decreased after treatment (Figure 5B), suggesting the reduction of STAT3 and NFAT was due to impaired gene transcription. Transcription of NFAT downstream target, such as interferon regulatory factor 4 (IRF4) also decreased. In contrast, *IL10*, which is up regulated by NFAT, did not change after treatment (Figure 5B).

We also measured BCL2 levels in DHL cells. When DOGKIT cells were treated with either compound, protein levels of BCL2 were reduced. A similar effect was observed in SU-DHL-6 cells when treated with high concentrations (Figure 5A). Consistently, the transcription of BCL2 were markedly decreased in both DOGKIT and SU-DHL-6 cells after either BBM or PA4 treatment (Figure 5B).

### 3.5 | CAMKII deletion similarly inhibits the growth of DHL cells

**FIGURE 6:**
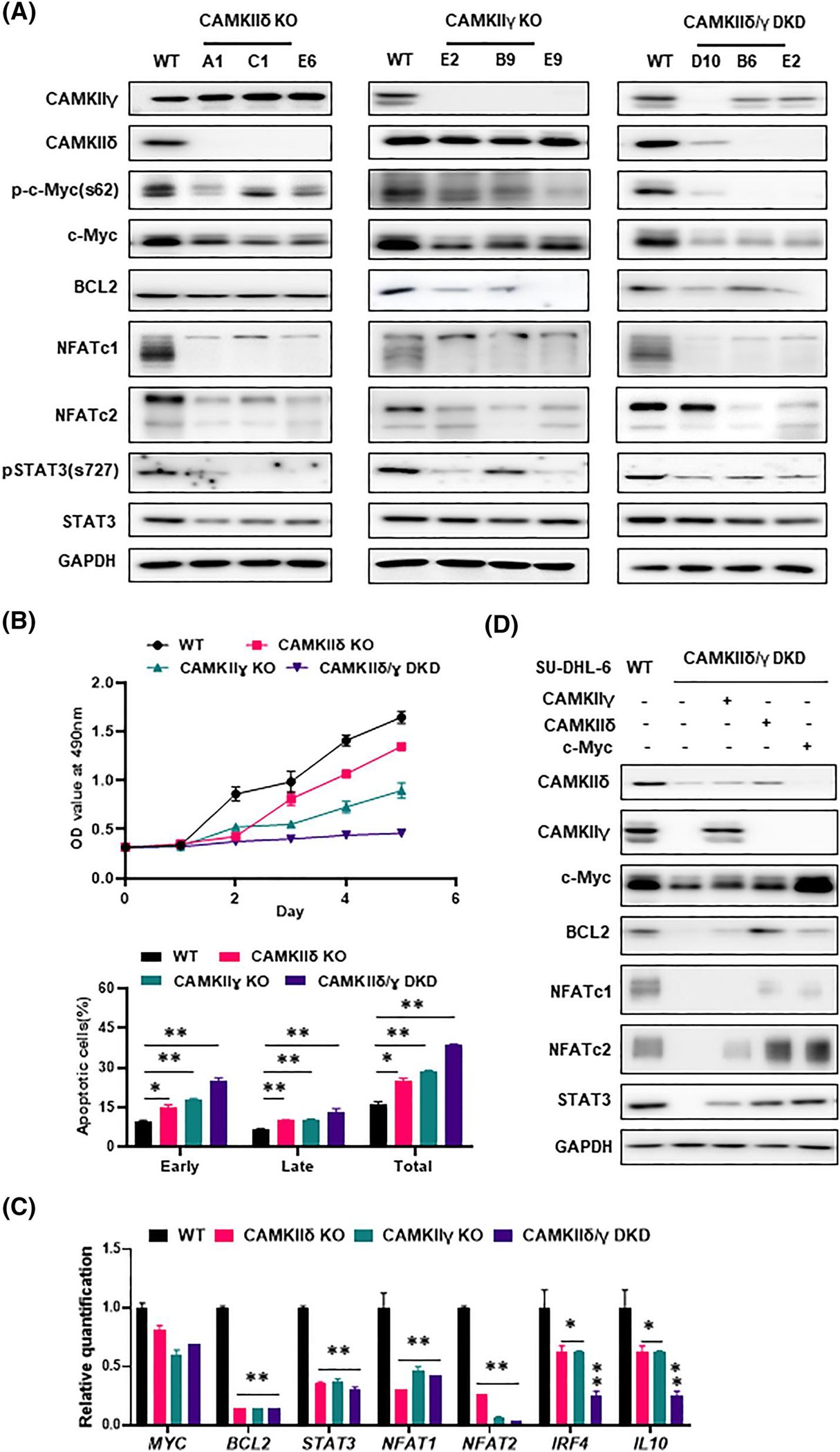
Deleting CAMKII affected biological features by decreasing the stability of c-Myc and impairing transcriptions of downstream targets. (A), Western blot analysis of CAMKII and potential targets in independent single cell clones of wide type (WT), Ca^2+^/calmodulin-dependent protein kinase II *δ* (CAMKIIδ) KO, Ca^2+^/calmodulin-dependent protein kinase II γ (CAMKIIγ) KO, and CAMKIIδ/γ DKO in SU-DHL-6. (B), Growth curve of WT and CAMKII deficient cells determined by MTS assay (upper panel). Percentages of apoptotic cells determined by flow cytometry. Cells were stained with Annexin V and DAPI and then evaluated on BD Fortessa cytometer. The collected data were analyzed by FlowJo software. (C), Exogeneous CAMKIIδ or CAMKIIγ or c-Myc was expressed by the transiently transfected plasmids in CAMKIIδ/γ DKD cell line and harvested cells 24 h after transfection. Western blot analysis. (D), mRNA levels of CAMKII deficient cells. Data are shown as mean ± SEM and significance are determined by unpaired Studenťs *t*-test. *p < 0.05 compared to WT; **p < 0.01 compared to WT

To better understand the impact of CAMKII in DHL, we used the CRISPR/Cas9 system to generate CAMKII knockout (KO) cell lines in SU-DHL-6. After single cell clone selection, we generated CAMKIIδ KO, CAMKIIγ KO, and CAMKIIδ/γ double knockdown (DKD) cell lines (Figure 6A). We failed to obtain CAMKIIδ/γ double knockout clones, suggesting that double knockout of CAMKIIδ/γ may be detrimental to cells.

Compared to the wild type (WT) parental cells, KO cell lines displayed lower proliferation capacity and higher apoptotic rate (Figure 6B and Figure S4A). KO cell lines were more resistant to either BBM or PA4 with higher IC_50_ (Figure S4B). KO cell lines exhibited mildly reduced *MYC* mRNA and significantly reduced *BCL2* mRNA. KO cell lines also significantly decreased mRNA levels of *STAT3*, *NFATc1*, and *NFATc2* (Figure 6C). Downstream target of NFAT pathway, *IL10* and *IRF4* mRNA were also downregulated (Figure 6C).

The decreased mRNA transcription in the KO cell lines (Figure 6C) correlated with reduced protein expression of c-Myc, STAT3, NFATc1, NFATc2, and BCL2 (Figure 6A). Both c-Myc and p-c-Myc (S62) were reduced in KO cell lines with double knockdown had a more pronounced reduction than single deletion. STAT3 and NFAT protein levels were reduced in all KO cell lines. BCL2 protein level was significantly decreased in CAMKIIγ KO and DKD cells. The impact of CAMKII deletion via CRISPR/Cas9 was consistent with pharmacological inhibition using either BBM or PA4.

Furthermore, rescue experiments were performed. We transiently transfected SU-DHL-6DKD cells with either CAMKIIδ, CAMKIIγ, or c-Myc plasmids. Exogenous expression of either CAMKIIδ, CAMKIIγ, or c-Myc partially rescued protein levels of BCL2, STAT3 and NFAT. Moreover, exogenous expression of either CAMKIIδ or CAMKIIγ upregulated c-Myc in SU-DHL-6 DKD cells (Figure 6D). These results suggest that the CAMKII-c-Myc axis is critical in DHL cell lines.

### 3.6 | PA4 exhibited a potent therapeutic effect on DHL in animal tumor models

**FIGURE 7:**
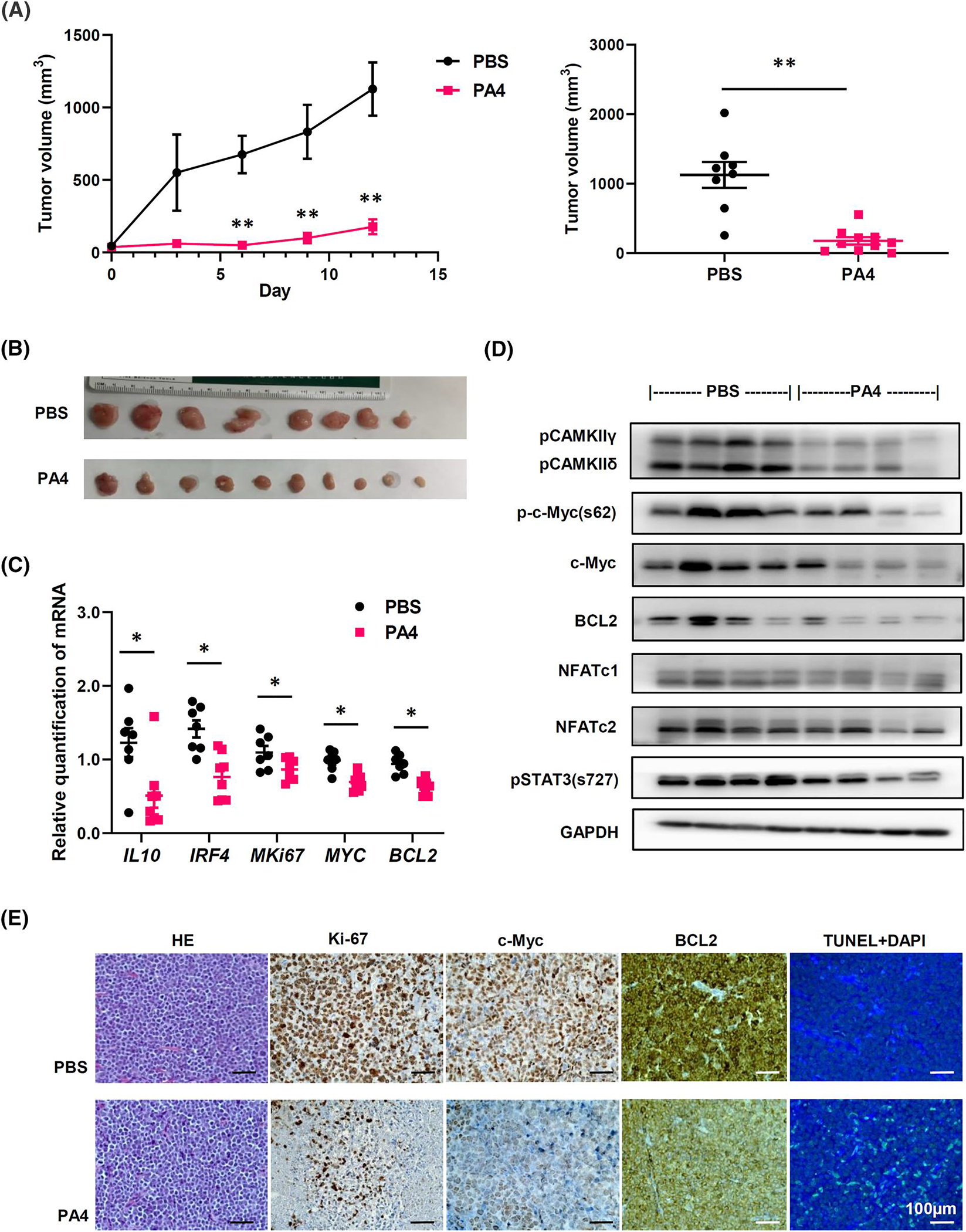
PA4 exhibited promising therapeutic effect in vivo. (A), Tumor growth curve in NSG mice treated with either PA4 or PBS for 12 days (left panel) and tumor size at endpoint of the 12^th^ day (right panel). (B), Tumors size on day 12. (C), mRNA level of *IL10*, *IRF4*, *MKI67*, *MYC* and *BCL2* in tumor tissues from PA4 and PBS groups. (D), Western blot analysis of CAMKII and potential targets in tumor tissues from PA4 and PBS groups. (E), Representative images of HE, Immunohistochemical (IHC), and TUNEL staining with sections of tumors from PA4 and PBS groups

To evaluate the anti-tumor effects of PA4 in vivo, we adopted a xenograft model using NSG mice inoculated with SU-DHL-6 cells. Single treatment of PA4 at a dosage of 5 mg/kg/d was sufficiently to control tumor growth in these animals (Figure 7A). When we euthanized the mice on day 12, tumor volume in PA4 group was markedly smaller than PBS group (Figure 7B).

Compared to PBS group, we observed reductions of *NFAT* mRNA and proteins (Figure 7C,D), with notable reductions of downstream *IL10* and *IRF4* mRNA. IHC staining showed weaker stained Ki67 in PA4 treatment group (Figure 7E). The STAT3 pathway, c-Myc and BCL2 were all strongly reduced after PA4 treatment (Figures 7D,E). Higher cell apoptosis was detected in the PA4 group as shown by TUNEL staining (Figure 7E).

## 4 | DISCUSSION

In DHL, characteristic gene rearrangements lead to overexpression of *MYC*/*BCL2* that promote tumor cell growth and survival.^14,15^ Patients with this unique subtype of lymphoma have poor outcomes.^2,16^ In this study, we tested the natural product BBM and its analog PA4^6,7,17,18^ as inhibitors of CAMKII in DHL. Our results indicate that CAMKIIδ is the dominant isotype expressed in DHL cell lines and is associated with high levels of c-Myc. Treatment with either BBM or PA4 can significantly inhibits DHL development in vitro and in vivo.

In our previous study on T cell lymphoma (6), we demonstrated that CAMKIIγ stabilizes c-Myc by directly phosphorylating it at Serine 62 (S62); this leads to cellular proliferation and lymphomagenesis.^19–21^ Our results in DHL here showed the same mechanism. Pharmacological inhibition of CAMKII with either BBM or PA4 impedes phosphorylation at S62 and results in degradation of c-Myc protein regardless of the high mRNA levels. This is critical because DHL has higher MYC transcription due to the *MYC* rearrangement. We observed similar c-Myc degradation when we genetically delete both CAMKIIδ/γ in SU-DHL-6.

Another potential target of CAMKII is the STAT3 pathway.^22,23,24^ Constitutive STAT3 activation is frequently observed in hematological malignancies,^25–29^ and has been associated with poor outcomes.^30–33^ STAT3 can be directly phosphorylated at S727 by CAMKII^24,34^, which is critical for STAT3 transcriptional activity. Furthermore, c-Myc is reported to be regulated by STAT3 pathway.^35^ Either BBM, PA4, or CAMKII deletion impeded STAT3 activation and may further contribute to c-Myc protein degradation.

Another important pathway in lymphomagenesis is NFAT pathway. NFAT pathway is chronically activated independent of BCR signaling and controls key biological processes in B cell lymphoma.^12,13,36,37^ The NFAT pathway was shown to be negatively regulated by CAMKII in a model of pathological cardiac myocyte hypertrophy.^38^ Our findings in DHL indicated that CAMKII inhibition lowered *NFAT* transcription and impaired NFAT activity. Interruption of the NFAT pathway in DHL cell lines may be indirectly induced by a reduction in c-Myc as either exogeneous CAMKIIδ or c-Myc could rescue the NFAT transcription and activity.

We also observed that in DHL, the CAMKII-Myc axis unexpectedly regulated the downstream protein BCL2. BCL2 is the hallmark anti-apoptotic protein overexpressed in DHL and a promising target for DHL therapy. Targeting BCL2 with ABT199 effectively inhibits cell growth in DHL.^3,4,39^ In this study, the combination of ABT199 with PA4 exhibited a moderate synergistic effect in vitro, which may result from concurrent inhibition of BCL2 and c-Myc. In a report by Wei et al., BCL2 was shown to be regulated by CAMKII.^40^ Here, we also observed that BCL2 transcription is decreased when CAMKII is inhibited. Accordingly, we observed lower levels of BCL2 protein in NSG models treated with PA4.

In summary, we have demonstrated a critical role of CAMKII in accelerating proliferation and inhibiting apoptosis in DHL. Although the potential off-target effects of BBM and PA4 need further verification and improvement for a future drug development, this study provides a proof-of-concept that targeting CAMKII by BBM and its derivative PA4 may lead to a promising DHL treatment using a single agent.

## Supplementary Material

Table S1

Table S2

Fig S4

Fig S2

Fig S3

Fig S1

## Figures and Tables

**TABLE 1:** Immunohistochemical (IHC) scores of clinical samples

No.	Cell origi-	CAMKIIδ	CAMKIIγ	p-c-myc (s62)	c-Myc	Bcl-2
1	Non-GCB	0	0	34	5%	+
2	Non-GCB	106.2	41.6	188.6	30%	-
3	Non-GCB	78.8	0	58.4	30%	+
4	Non-GCB	32.8	0	50.8	10%	+
5	Non-GCB	10.8	5.6	7.4	-	-
6	Non-GCB	53.8	0	22.8	10%	+
7	Non-GCB	1.6	0.6	5.6	20%	+
8	Non-GCB	0	0	0	-	+
9	Non-GCB	78.2	0	0	10%	60%
10	Non-GCB	150.4	0	106.8	50%	50%
11	Non-GCB	8.6	10.4	12.6	20%	60%
12	GCB	183.6	87.4	0	30%	-
13	Non-GCB	127	1.4	84.6	40%	-
14	GCB	114	21.4	9.2	10%	+
15	GCB	34	21.4	9.2	30%	70%
16	Non-GCB	2	0.6	12.4	20%	90%
17	Non-GCB	97.6	92.2	67.4	50%	-
18	Non-GCB	45.2	4.6	33	20%	90%
19	GCB	124.8	107.4	158.2	60%	-
20	GCB	112.2	84.8	52.6	40%	50%
21	DHL	181.4	82.6	177.8	-	-
22	GCB	55.4	53.8	187.6	-	-
23	Non-GCB	11.2	0	63.4	50%	-
24	GCB	67.8	0	112	70%	50%
25	Non-GCB	118	0	96.6	40%	+
26	GCB	105.4	0	69.8	60%	30%
27	Non-GCB	0	0	0	40%	70%
28	GCB	31	17.2	64.4	50%	80%
29	DHL	113.4	0	85.2	40%	-
30	GCB	170.8	42.6	61	40%	-
31	DHL	218	125.4	147.2	-	+
32	DHL	240.6	164.4	186		
33	DHL	162	134.8	198.4	90%	+
34	DHL	190.4	117	185.6	90%	+
35	DHL	1.5	157.8	0	-	-
36	DHL	158.8	196.2	149	+	+
37	DHL	95.6	41	137.2	90%	-
38	DHL	182.4	104	125.8	-	+

*Note:* GCB: *n* = 10; Non-GCB: *n* = 18; DHL: *n* = 10.

## Data Availability

All data, models, and code generated or used during the study appear in the submitted article.
