# Supplementary material for: Targeting MYC and BCL2 by a natural compound for “double-hit” lymphoma
Source: Hematol Oncol. Author manuscript; Available in PMC 2022 Aug 16. (PMC9378491; doi:10.1002/hon.3010)
Supplement: Table S1 [file NIHMS1807194-supplement-Table_S1.docx]

| **Suppl. Table 1** | **antibodies used for IHC and WB** |  |
| --- | --- | --- |
| **Antibodies** | **Source** | **Catalog No.** |
| Anti-CAMKIIg (C-18) | Santa Cruz Biotechnology | sc-1541 |
| Anti-CAMKIIg (8G10C1) (IHC) | Abcam | ab201966 |
| Anti-CAMKIId(EPR13095) | Abcam | ab181052 |
| Anti-CAMKII(D11A10) | Cell Signaling Technology | 4436 |
| Anti-Phospho-CAMKIIg | Santa Cruz Biotechnology | sc-12886-R |
| Anti-Phospho-CAMKII (Thr286) (D21E4) | Cell Signaling Technology | 12716S |
| Anti-c-Myc (Y69) | Abcam | ab32072 |
| Anti-Phospho-c-Myc (Ser62) (E1J4K) | Cell Signaling Technology | 13748 |
| Anti-Phospho-STAT3(Ser 727) | Cell Signaling Technology | 9134S |
| Anti-STAT3(124H6) | Cell Signaling Technology | 9139S |
| Anti-Bcl2(D55G8) | Cell Signaling Technology | 4223S |
| Anti-NFATc2(NFAT1) | Santa Cruz Biotechnology | sc-136206 |
| Anti-NFATc1(NFAT2)(7A6) | Santa Cruz Biotechnology | sc-7294 |
| Anti-GAPDH | Cell Signaling Technology | 2118 |
| Anti-Flag | Cell Signaling Technology | 8146S |
| Anti-HA(12CA5) | Roche | 11666606001 |
| Anti-rabbit IgG, HRP-linked Antibody | Cell Signaling Technology | 7074S |
| Anti-mouse IgG, HRP-linked Antibody | Cell Signaling Technology | 7076S |
