# Supplementary material for: Targeting MYC and BCL2 by a natural compound for “double-hit” lymphoma
Source: Hematol Oncol. Author manuscript; Available in PMC 2022 Aug 16. (PMC9378491; doi:10.1002/hon.3010)
Supplement: Table S2 [file NIHMS1807194-supplement-Table_S2.docx]

| **Suppl. Table 2** |  |  |
| --- | --- | --- |
| **Primers for real time RT-PCR** | | |
| *CAMK2A* | Forward | GATGGCAAATGGCAGATCG |
|  | Reverse | ATCTCTGCGGCACAGCAAC |
| *CAMK2B* | Forward | CCAGCCAGTCTGAGGAGACC |
|  | Reverse | GGCTCCAAACACCAACTCTGT |
| *CAMK2G* | Forward | TGGCTCAATGTCCACTATCACTG |
|  | Reverse | AAACCTCAAACAAACAGGACTGC |
| *CAMK2D* | Forward | ACTTGTGGGGTATCCACCCTTC |
|  | Reverse | TCAAGCAGTCTACAGTCTCCTGTC |
| *MYC* | Forward | TGCAGCTGCTTAGACGCTGGATTT |
|  | Reverse | GTCGAGGTCATAGTTCCTGTTGGT |
| *BCL2* | Forward | GTGAACTGGGGAGGATTGT |
|  | Reverse | GTGCCGGTTCAGGTACTCAG |
| *STAT3* | Forward | CAGCAGCTTGACACACGGTA |
|  | Reverse | AAACACCAAAGTGGCATGTGA |
| *NFAT1* | Forward | GGCTGTCAAAGCTCCAACTG |
|  | Reverse | GTCCCAGAGGCTTGTTTTCC |
| *NFAT2* | Forward | CCGACATTGAACTTCGGAAA |
|  | Reverse | GGATAGCTGTCCGTGCTCTG |
| *IRF4* | Forward | GCCAGCTGGACATCTCAGAC |
|  | Reverse | AGGCGTTGTCATGGTGTAGG |
| *IL10* | Forward | GCACCCAGTCTGAGAACAGC |
|  | Reverse | CTCAGACAAGGCTTGGCAAC |
| *MKi-67* | Forward |  |
|  | Reverse |  |
| *GAPDH* | Forward | TCATTGACCTCAACTACATG |
|  | Reverse | TCGCTCCTGGAAGATGGTGAT |
| **sgRNA for Crispr** |  |  |
| *CAMK2G-crispr-e1* | Top | CACCGCTCGAAGAGCTGGTAGTCGT |
|  | Bottom | AAACACGACTACCAGCTCTTCGAGC |
| *CAMK2D-crispr-e7* | Top | CACCGGCTTAGCCATAGAAGTTCAA |
|  | Bottom | AAACTTGAACTTCTATGGCTAAGCC |
| **Primers for single cell clone selection** | | |
| *CAMK2G* | Forward | CCGTCTCCTCCTCTTGCTC |
|  | Reverse | TACTTGCCAAGCTCCTCGAA |
| *CAMK2D* | Forward | TAGCAAATCCAAGGGAGCAG |
|  | Reverse | ATATCCAGGTGTGCCAGCAA |
