# Supplementary figures and images for: Targeting MYC and BCL2 by a natural compound for “double-hit” lymphoma
Source: Hematol Oncol. Author manuscript; Available in PMC 2022 Aug 16. (PMC9378491; doi:10.1002/hon.3010)

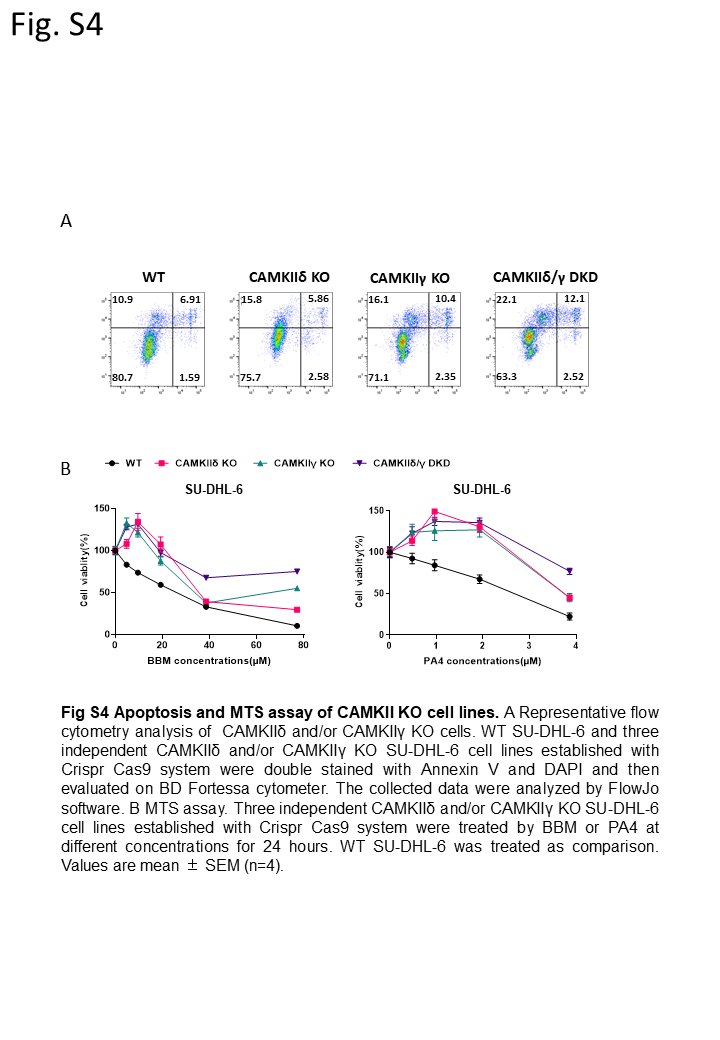

Supplement: Fig S4 [file NIHMS1807194-supplement-Fig_S4.tif]

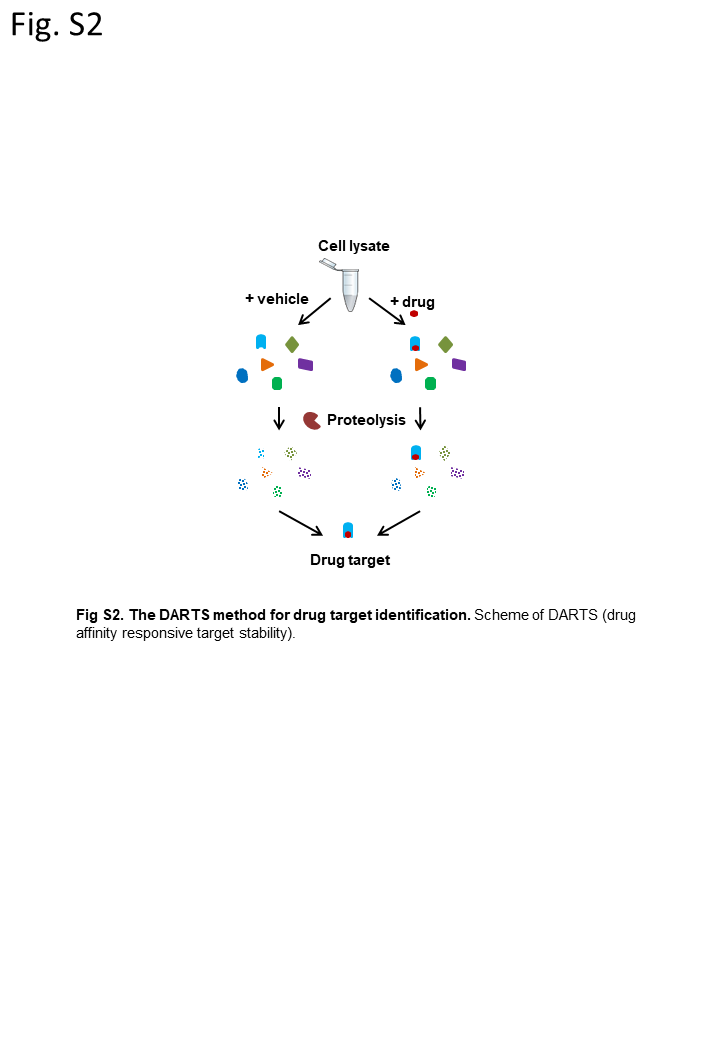

Supplement: Fig S2 [file NIHMS1807194-supplement-Fig_S2.tif]

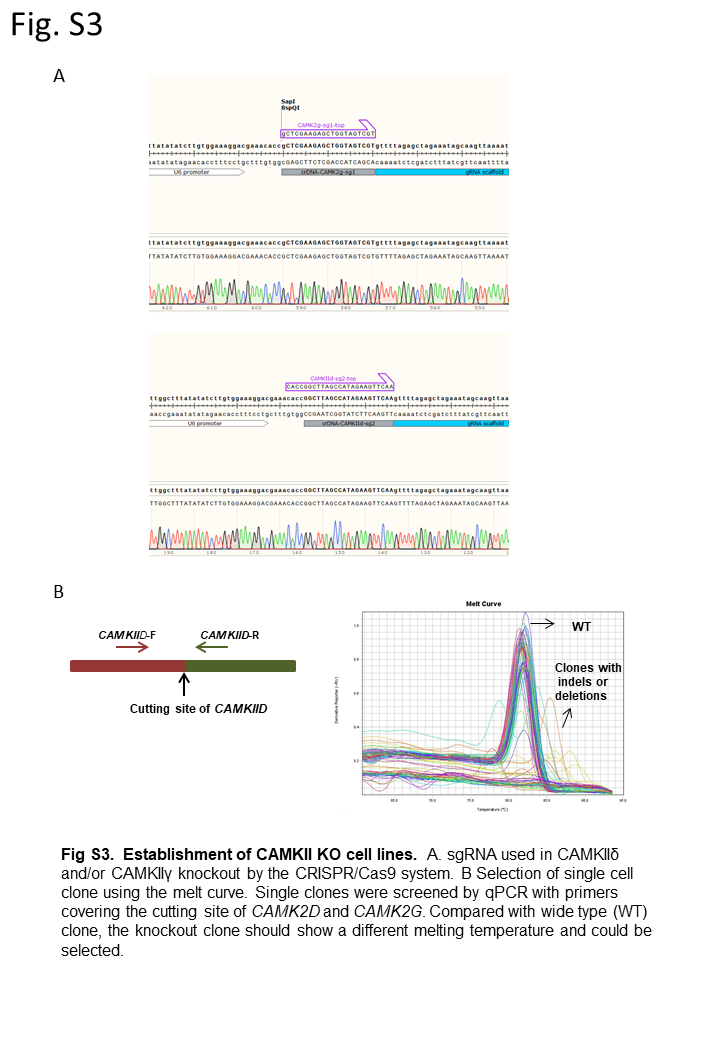

Supplement: Fig S3 [file NIHMS1807194-supplement-Fig_S3.tif]

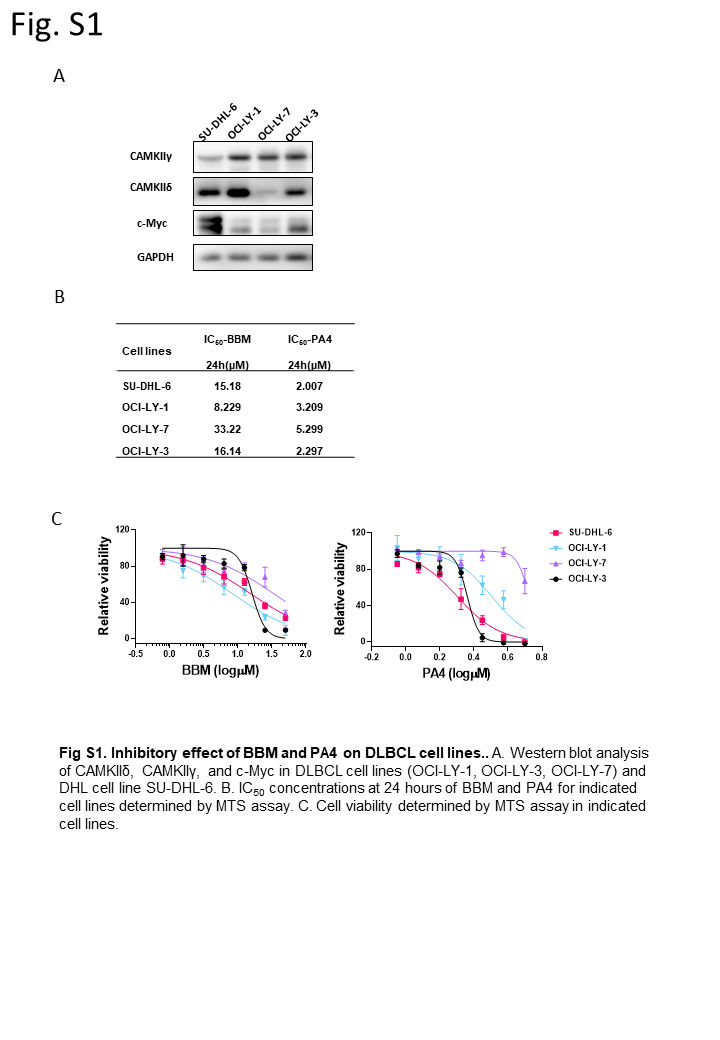

Supplement: Fig S1 [file NIHMS1807194-supplement-Fig_S1.tif]
